# Formulation and Stability of a 1% Clarithromycin-Based Topical Skin Cream: A New Option to Treat Buruli Ulcers?

**DOI:** 10.3390/ph17060691

**Published:** 2024-05-27

**Authors:** Maria Sebti, Arnaud Schweitzer-Chaput, Salvatore Cisternino, Mélanie Hinterlang, Dimitri Ancedy, Sandrine Lam, Sylvain Auvity, Camille Cotteret, Olivier Lortholary, Joël Schlatter

**Affiliations:** 1 Service Pharmacie, Hôpital Universitaire Necker-Enfants Malades, Assistance Publique des Hôpitaux de Paris (APHP), 149 Rue de Sèvres, F-75015 Paris, France; maria.sebti@aphp.fr (M.S.); arnaud.schweitzerchaput@aphp.fr (A.S.-C.); melanie.hinterlang@aphp.fr (M.H.); dimitri.ancedy@aphp.fr (D.A.); sandrine.lam@aphp.fr (S.L.); sylvain.auvity@aphp.fr (S.A.); camille.cotteret@aphp.fr (C.C.); joel.schlatter@aphp.fr (J.S.); 2 Faculté de Pharmacie, Université Paris Cité, Inserm UMRS 1144, 4, Avenue de l’Observatoire, F-75006 Paris, France; 3 Service des Maladies Infectieuses et Tropicales, Hôpital Universitaire Necker-Enfants Malades, Assistance Publique des Hôpitaux de Paris (APHP), F-75015 Paris, France; olivier.lortholary@aphp.fr; 4 Institut Pasteur, Molecular Mycology Unit, National Reference Centre for Invasive Mycoses and Antifungals, CNRS UMR 2000, F-75015 Paris, France; 5 Institut Imagine, Hôpital Universitaire Necker—Enfants Malades, F-75105 Paris, France; 6 Service Pharmacie, Hôpital Paul Doumer, Assistance Publique des Hôpitaux de Paris (APHP), 1 Rue de l’Hôpital, F-60140 Labruyère, France

**Keywords:** Buruli ulcers, clarithromycin, drug compounding, macrolide, tropical disease

## Abstract

There are more than 170 known species of non-tuberculous mycobacteria, and some are responsible for serious diseases in people infected with them. One of these is Buruli ulcers, a neglected tropical disease endemic in more than 33 countries and caused by *Mycobacterium ulcerans*, which infects skin tissue. Treatment consists of a long-term regimen combining the use of oral rifampin with another anti-tuberculosis drug (e.g., clarithromycin). Patients in these countries face difficulties in accessing and adhering to this therapy. This study investigates the feasibility of formulating stable, optimized clarithromycin as a topical cutaneous cream. The cream was formulated, and its stability was evaluated under different storage temperature conditions and using a stability indicator method. The results showed that the clarithromycin cream was stable for at least 60 days, even at extreme temperatures (40 °C). In conclusion, the data presented here demonstrate the stability of a new form of topical cutaneous clarithromycin, which may offer a new approach to the treatment of Buruli ulcers and clarithromycin-sensitive infections.

## 1. Introduction

Infection with non-tuberculous mycobacteria, particularly *Mycobacterium marinum*, *Mycobacterium ulcerans* (*M. ulcerans*), and *Mycobacterium leprae*, is a serious global public health problem affecting mainly subtropical and tropical areas. Such infections may be associated with a variety of contamination routes, including contaminated water, exposure to contaminated animals, or the environment, although these routes and vectors remain unclear [1,2]. Clinical symptoms are diverse and heterogeneous, but usually involve cutaneous lesions including papules, nodules, ulcers, and edema, which can lead to scarring, deformity, and amputation. *M. ulcerans* is the bacterium that causes Buruli ulcers, which is one of the world’s most neglected tropical diseases and the third most common human mycobacterial disease [3,4,5].

**Figure 1:**
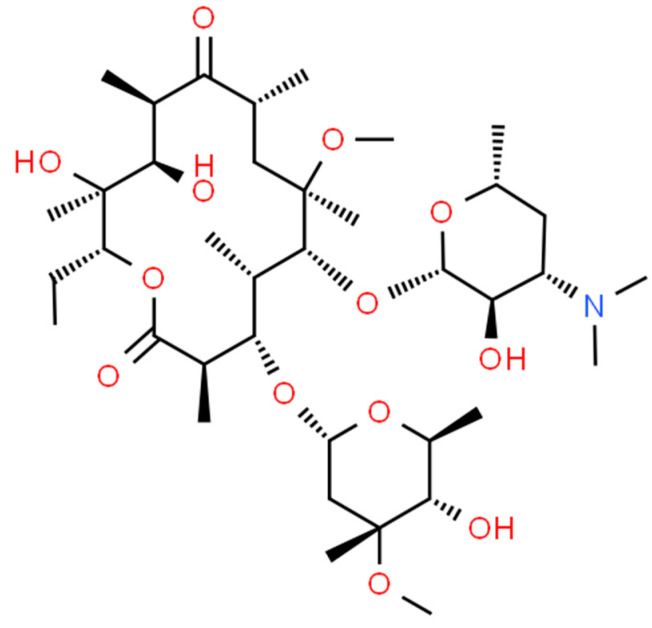
Chemical structure of clarithromycin.

Treatment of Buruli ulcers remains difficult and may involve a combination of antibiotics and possible surgery [6]. If the infection recurs, then oral medication may be required for several months or years [7,8,9]. Systemic therapy with rifampicin, isoniazid, and ethambutol has traditionally been the standard of care. More recently, streptomycin, moxifloxacin, and macrolides such as azithromycin and clarithromycin (CLA) have been introduced to treat such infections in combination with rifampicin. Indeed, CLA has some interesting properties for treating such infections. CLA, also known as 6-O-methyl-erythromycin (Figure 1), is a semi-synthetic macrolide antimicrobial that is widely used to treat respiratory, skin, and soft tissue infections [10,11]. It is a broad-spectrum antibiotic, active against a wide range of Gram-positive and Gram-negative microorganisms, including atypical ones such as mycobacteria [12].

Much effort is being put into the introduction and evaluation of new therapies that may have the potential to improve efficacy and compliance in the treatment of Buruli ulcers [5]. The social dimension and cultural aspects in the regions concerned also affect the chances of treatment success [13,14]. Cost, the availability of treatments, and, in particular, the need for compliance over such long periods of therapy are major obstacles that affect the chances of achieving a satisfactory outcome [3,4,15]. Recovery is slow and the recurrence of infection is common [2,3,16].

Some research has also suggested that adjunctive treatment with topical creams may be beneficial in the treatment of skin lesions. Such treatments can help to reduce the bacterial load, heal the lesions, and prevent the local spread of infection. The use of topical antimicrobials as an adjunct or not to systemic treatment can be decided on an individual basis, taking into account various parameters such as the size, localization, and staging of the skin lesion [17,18,19].

In agreement with our travel medicine institutes and our medical institutions, the aim is to develop a topical skin form of CLA that could be produced by a hospital pharmacy and whose relatively long shelf life would also facilitate logistics adapted to overseas territories (e.g., French Guyana). Although the use of erythromycin to treat acneiform lesions is well accepted, CLA does not currently have a topical formulation for the skin, apart from a study for corneal use [20]. The aim of this study is to develop a formulation of 1% CLA cream for topical skin application and to evaluate its physical and chemical stability over time.

## 2. Results

### 2.1. Validation of the Stability-Indicating HPLC Method

**Figure 2:**
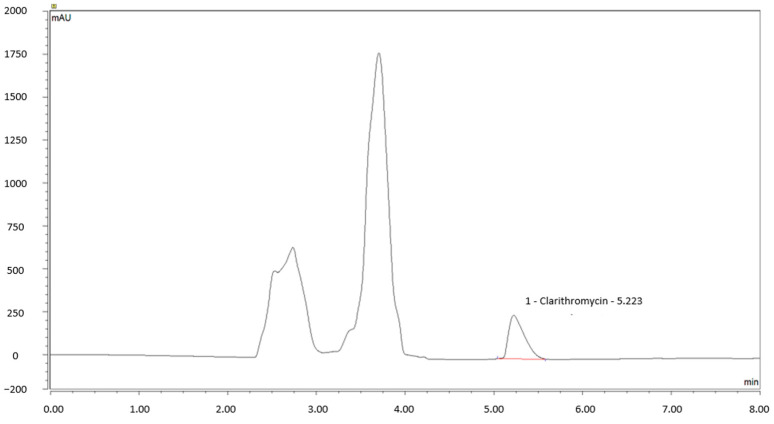
Typical clarithromycin cream (CLA) chromatogram showing peaks associated with Pentravan^®^ well separated from those of CLA (~5.2 min).

This method has been validated by assessing performance specifications related to specificity, linearity, LOD, LOQ, precision, accuracy, and robustness. The linearity of the method has been demonstrated over the range of 0.8 to 1.2 mg/mL. The calibration curve equation in the cream was y = 35.31x (±0.36) − 3.71 (±0.36) and r^2^ = 0.998; in water, y = 39.55x (±0.78) − 0.01 (±0.81) and r^2^ = 0.999. The details of the results are summarized in Table 1. A typical chromatogram obtained for this 1% CLA cream formulation after extraction with methanol is shown in Figure 2.

The determined values of LOD and LOQ were 0.05 and 0.14 mg/mL, respectively. The intra and inter-day precision for the determination of CLA were summarized in Table 2. For every determination, the RSD values were less than 0.4%. The results of the RSD studies demonstrated the precision of the proposed method.

The percentage recoveries were spread from 100.3% (±0.3) to 101.5% (±0.3) with RSD values in the range of 0.3% to 0.9%. The results of the recovery studies showed the accuracy of this method. Details of the results are summarized in Table 3.

A limitation of this study was the extraction of CLA in the cream. Different extraction solvents were studied, such as acetone, acetonitrile, and methanol (Table 4). The most suitable extraction solvent was methanol. Acetonitrile extraction was promising, but coelution occurred, making CLA dosing unfeasible. After methanol extraction, drug recovery of 1% CLA cream was 80.1% (±0.6; n = 9), regardless of the concentration tested between 0.8 and 1.1% of CLA.

The forced degradation studies demonstrated that CLA is sensitive to both acid and oxidative stress. Indeed, CLA was immediately and entirely degraded after an hour in acidic conditions. In oxidative stress conditions, only 14% of CLA remained intact after one hour. Nevertheless, the CLA appeared stable under alkaline conditions. These results are shown in Table 5.

### 2.2. Physico-Chemical Stability Study

**Figure 3:**
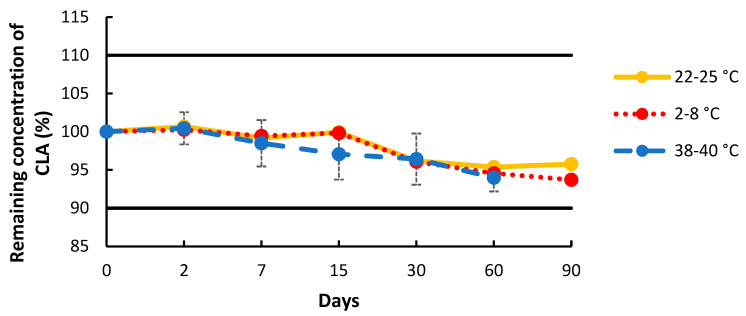
Stability over time of formulated 1% clarithromycin (CLA) cream at 2–8 °C (red dotted line), 22–25 °C (yellow solid line), and 38–40 °C (blue dashed line) in plastic Topitec jar. CLA residual concentration, calculated as the ratio of the concentration on the day tested to the concentration on day 0; data are mean ± SD (n = 3).

The percentage of remaining CLA concentration during the storage time is shown in Figure 3. The 1% CLA cream was found to be stable over a 90-day period when stored in a Topitec^®^ jar at room temperature and under refrigeration. The 1% CLA cream when stored at 38–40 °C showed chemical stability for 60 days.

In terms of physical stability, the cream appeared to be smooth and odor-free throughout the duration of the study, at both room and refrigerated temperatures. The spread of the cream on skin was slightly oily, but skin absorption was fast with a dry skin touch. At high temperatures of storage (38–40 °C), the cream separated into two phases by day 15 and became liquid. The mixing of these two phases sufficed to bring the cream back to its initial consistency. No detectable change in color occurred. On day 90, the CLA cream stored at 38–40 °C was no longer chemically stable (<90%).

**Figure 4:**
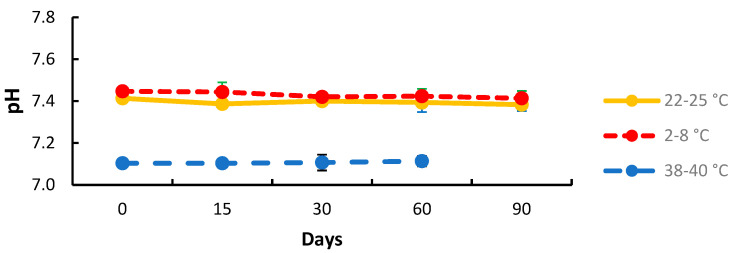
pH value of 1% clarithromycin cream stored over time at 2–8 °C (red line), 22–25 °C (yellow solid line), and 38–40 °C (blue line) in plastic Topitec jar (standard deviation bars are not visible on the figure due to low data scatter; n = 3).

The pH of the 1% CLA cream was not statistically different over time in any of the three storage conditions (Figure 4; *p* > 0.05).

## 3. Discussion

The social conditions and medical deserts of certain endemic regions can make the care of infected populations difficult. In addition, oral or systemic treatments are more likely to have adverse effects and may be more restrictive in their use. The use of locally available treatments can help to improve the quality of care and even to increase adherence to treatment. Finding new local therapies, particularly for Buruli ulcers, represents a real public health advance, aiming to provide new treatment options for affected tropical and subtropical populations. CLA appears to be an interesting alternative to other drugs for the treatment of these cutaneous mycobacterial infections [7,21,22]. Oral CLA has been shown to be effective in the treatment of some cutaneous mycobacterial infections. Its mechanism allows CLA to concentrate in infected skin cells. Consequently, it is possible to treat the intracellular microorganisms *M. ulcerans* or *Mycobacterium leprae* [23,24]. The topical application of a skin therapy may be an additional treatment for cutaneous mycobacteriosis associated with these non-tuberculous mycobacteria. The topical preparation of CLA may improve antibiotic delivery to the skin site(s) and optimize treatment adherence.

The primary aim of using an antibiotic cream is to treat as close to the infected area as possible. In addition, the topical route prevents both systemic absorption and toxicity [25,26,27]. By formulating a cream containing CLA, our aim is to target the deep layers of the dermis without achieving a systemic effect. This CLA cream is intended for use in the early stages of infection, particularly in the form of papules or nodules, and possibly in patients who are unwilling or unable to take oral treatment. Based on anti-mycobacterial susceptibility testing, the minimum inhibitory concentration (MIC) of CLA varies between 0.25 and 8 µg/mL [4,28]. At these MIC levels, a 1% topical form of CLA would be expected to have a pharmacological effect and thereby suppress the growth of the strain. To this end, we have developed a topical 1% CLA cream.

Advances over the past decade have made it possible to offer a range of ready-to-use skin cream bases. This has made the formulation of topical skin medicines more accessible and convenient to manufacture, particularly for hospital pharmacies. Among these bases, Pentravan^®^ is an oil-in-water emulsion with a liposomal matrix and a variety of permeation enhancers. It has shown improved skin penetration for many active ingredients [29,30,31]. Unlike Pluronic Lecithin Organogel (PLO) and other lipophilic bases, Pentravan’s true disappearance and fast permeability means it leaves no sticky residue on the skin and, most importantly, does not require an occlusive dressing after application, making it easier for people to comply and for communities to use it. Erythromycins like CLA are poorly soluble in water [32]. The solubility values of erythromycin in various chemical penetration enhancers reported in the literature show that the solubility in Transcutol^®^ is the best and guided us in our choice to allow for a complete solubilization of the CLA prior to incorporation into Pentravan^®^ [20,33,34,35]. As well as its ability to solubilize hydrophilic and lipophilic active ingredients, Transcutol^®^, a diethylene glycol monoethylether, was chosen to improve skin penetration when diluted in water, and due to application safety [33].

To assess the need for excipients to chemically stabilize the CLA in the cream, forced drug degradation was performed. CLA was found to be sensitive to oxidizing conditions and acidic pH [36,37,38]. As an antioxidant, ascorbic acid was added to the formulation. The pH of the final formulation was adjusted to ~7.3 to limit CLA degradation. In fact, other studies confirm that CLA degrades more rapidly at low pH [37,38]. One study reported a 25% degradation at pH 2.0 in 30 min of incubation, while at pH 1.5, 70% of the drug was degraded in the same time [38]. Interestingly, the inhibitory effect of CLA depends on the medium pH; the drug is more active at pH 7.4, but less at pH 5.0, with pH 6.8 being intermediate. The neutral pH chosen makes it possible to optimize the chemical stability and pharmacological action of the CLA, as well as its acceptance by the skin. Topitec^®^ touch has also been used for the production of semi-solid formulations in pharmacies. This system reduces external contact and the risk of contamination by inserting fingers into the preparation. It ensures efficient, homogeneous, and reproducible mixing [39].

An analytical HPLC method was developed and validated according to ICH Q2 to study and assess the physico-chemical stability of the CLA cream [40]. Physico-chemical stability was demonstrated for the formulated 1% CLA cream for 90 days at room and refrigerated temperatures and for 60 days at 39 ± 2 °C. As a result, this formulation has been optimized to prevent potential natural degradation processes. This study demonstrated that a cream formulation of 1% CLA is feasible and could be used for cutaneous therapy.

By analogy with its counterpart, a schedule similar to that used for topical erythromycin treatment with a twice-daily cream can be considered for treating the skin with CLA. The use of topical erythromycin on damaged skin is not recommended. Similarly, only non-necrotic stages of the disease should be treated with this non-sterile CLA cream. The safety of this cream on damaged skin is not known, so any use in an ulcerative stage of the disease should be avoided. Ultimately, this topical treatment could play a supportive role in wound care to achieve the best healing outcome in the shortest time. Therefore, early detection and prompt treatment are essential in order to achieve an effective outcome and to minimize the disability associated with the advanced stages of NTM.

The development of a topical skin formulation of CLA may help to address the limited therapeutic options for the treatment of cutaneous manifestations of NTM infections. To our knowledge, this is the first report to investigate the stability of CLA cream. This study demonstrates the feasibility of a physico-chemically stable CLA cream, allowing for the further characterization of CLA skin permeability ex vivo and efficacy in vivo.

## 4. Materials and Methods

### 4.1. Chemical and Reagents

Analytical-grade CLA powder was provided by Merck (Saint Quentin Fallavier, France). The pharmaceutical CLA powder was provided by Mylan (Lyon, France) and is composed of clarithromycin lactobionate (Zeclar^®^, Paris, France, batches 88678TB23, 09096TB23, 04532TB26, and 04533TB25). Transcutol^®^ (diethylene glycol monoethylether, Thiais, France, batch 190130A) and Pentravan^®^ (Thiais, France, batch 1908979; ingredients: polyoxyl 40 stearate, potassium sorbate, zemea USP-NF, propanendiol, carbomer homopolymer type C, purified water, edetate disodium dihydrate, benzoic acid, sorbic acid, lecithin, isopropyl myristate, simethicone, isopropyl palmitate, cetyl alcohol, glyceryl monostearate, stearyl alcohol, stearic acid, butylated hydroxytoluene, and hydrochloric acid) were provided by Fagron, Thiais, France. Pharmaceutical-grade ascorbic acid (Melun, France, batch 17050189/A03) was provided by Cooper. Purified water was obtained from Fresenius Kabi (Versylene^®^, Sèvres, France). All chemicals and reagents were analytical-grade. Methanol, acetonitrile, and hydrochloric acid were provided by VWR (Fontenay-sous-Bois, France). Sodium hydroxide was provided by Cooper. Hydrogen peroxide was provided by Gilbert (Herouville Saint-Clair, France). Potassium phosphate monobasic (Darmstadt, Germany) was provided by Merck.

### 4.2. CLA Cream Formulation

The final optimized formulation of 1% CLA cream (*w*/*w*) is shown in Table 6 and was prepared by firstly dissolving an appropriate quantity of CLA powder (as a lactobionate salt) in Transcutol^®^. Transcutol^®^ was used as a co-surfactant and penetration enhancer. It is a highly purified solvent used for active ingredients with low solubility in water, such as CLA. Pentravan^®^ is a proprietary oil-in-water vanishing cream topical vehicle for compounding, which was added to this preparation of CLA and Transcutol^®^, followed by ascorbic acid. All compounds were introduced into one empty Topitec^®^ polypropylene pot with a volume capacity of 100 g/125 mL (Fagron, Thiais, France), and then automatically stirred over 15 min with the Topitec^®^ Touch system (Hillscheid, Germany). This technology enables the homogeneous preparation of semi-solid cream in a closed system through vertical and circular movements using a closed rotary disc system. The pH of the mixture was adjusted to 7.3 ± 0.2.

### 4.3. Validation of the Stability-Indicating Liquid Chromatography Assay

#### 4.3.1. Chromatographic Conditions

The stability-indicating HPLC method used for analyzing CLA was developed and adapted based on previously published methods [41,42,43]. The HPLC system (Dionex^®^ Ultimate 3000, ThermoScientific, Villebon, France) comprised an RS-3000 degasser, an HPG-3000 pump, a WPS-3000 auto sampler, a column oven, and an RS-3000 diode array detector (DAD). Data acquisition (e.g., absorbance, peak time, area) was carried out using in-line Chromeleon^®^ software (V8.0, Thermo-Fisher, Villebon, France). A C18 column (Polaris^®^, 250 × 4.6 mm; particle size 5 μm; Agilent, Courtaboeuf, France) with in-line filter (10 µm; Interchim, Montluçon, France) was used and maintained at 50 °C. The flow rate was set at 1.0 mL/min. Isocratic elution was performed using acetonitrile and 0.05 M phosphate buffer pH 3.5 (45:55, *v*/*v*) as the mobile phase. The ultraviolet (UV) absorbance wavelength was set at 210 nm for quantification. The sample injection volume was set at 50 µL.

#### 4.3.2. Validation of the HPLC Method

The HPLC method, consisting of a 5-standard calibration curve (0.8 to 1.2 mg/mL), was used for CLA quantification, and has been validated for linearity, specificity, limits of detection (LOD) and quantification (LOQ), precision, and accuracy according to ICH Q2 validation guidelines [44]. By diluting CLA in distilled water, two stock solutions of CLA were prepared daily for three days to independently prepare five calibration standards and three quality controls. Linearity was studied by calculating the slope, intercept, and regression coefficient (r^2^). Accuracy was determined by means of quality control checks and is expressed in terms of percentage recovery, with ±5% as the acceptance criteria, by the following equation:

$$Accuracy=\frac{Experimental\;concentration}{\begin{array}{c} Theoritical\;concentration \end{array}}\times 100\%$$

The limit of detection (LOD) and limit of quantification (LOQ) for the CLA assay were determined by calibration curve method by using the following equations:

$$LOD=3.3\times \frac{s(intercept)}{s(slope)},$$

$$LOQ=10\times \frac{s(intercept)}{s(slope)},$$

where s(intercept) and s(slope) are the standard deviation of the y-intercept and slope of the calibration curve, respectively.

Precision was investigated with intraday and interday assays. The intraday precision was determined by measuring three independent quality control samples of CLA, injected six times on the same day. The interday precision was estimated by injecting quality control samples prepared at the same concentrations on three different days. The results were reported in terms of relative standard deviation (RSD). The accuracy was investigated by using the standard addition method at different levels (80, 90, 100, 110, and 120%). The mean recovery of CLA of the target concentration (1 mg/mL) was calculated and accepted with 100 ± 2%.

Method selectivity was ensured so that no excipient or degradation product chromatographic peak coincided with that of CLA. Chromatograms in the presence of all excipients were visually examined for changes in CLA peak shape and peak purity using the DAD detector scan from 200 to 300 nm. To study the ability of the method to detect CLA degradation products, the CLA cream was subjected to various forced degradation conditions. Thirty grams of 1% CLA cream were mixed with sodium hydroxide solution (5 M), hydrochloric acid solution (5 M), and 3% hydrogen peroxide maintained at 60 °C for 48 h.

#### 4.3.3. Sample Preparation

One gram of CLA cream sample was added to 5 mL of methanol in a glass vial and shaken for 3 h. The mixture was then centrifuged at 10,900 rpm for 15 min. The supernatant was collected and filtered using a cellulose filter (0.2 µm, Interchim). To ensure the method’s repeatability, fresh QC samples were analyzed each day prior to the determination of the CLA content for the stability study.

### 4.4. Chemical Stability of the Topical Formulation

The stability of the 1% CLA cream was assessed according to the ICH guidelines for the stability testing of new drug substances and products [44]. Three 1% CLA creams stored in a Topitec^®^ cream jar were tested at each of the selected temperatures: 5 ± 3 °C, 25 ± 3 °C, and 39 ± 2 °C. The chemical stability of the drug was assessed following preparation (day 0) and on days 2, 7, 15, 30, 60, and 90. The chemical stability of the compounded drug was based on a content of the drug not less than 90% and not more than 110% of the stated amount on the label [40,45].

### 4.5. Physical Stability

The physical properties of the product were investigated by the visual inspection of stored products under every condition. The color, odor, texture, appearance, and pH were evaluated on days 0, 7, 15, 30, 60, and 90. The samples’ pH value was determined each day of the study using an InLab^®^ Viscous Pro ISM model pH meter that was calibrated each day (Mettler-Toledo, Viroflay, France). The results are expressed as the mean ± standard deviation (SD).

### 4.6. Statistical Analysis

Data analysis was carried out with Prism^®^ software (version 6.01, GraphPad^®^ Software, San Diego, CA, USA). The descriptive statistics of the continuous variables are expressed as the mean ± SD.

## 5. Conclusions

The 1% CLA cream is stable for at least 90 days when stored in the refrigerator and at room temperature. It was also stable at high temperatures for at least 60 days. This preparation provides a new form of antibiotic that is available and convenient for the treatment of skin infections caused by Mycobacterium species. This is aimed at optimizing patient management and providing a new adjunctive option to treat and prevent the recurrence of cutaneous manifestations of NTM.

## Figures and Tables

**Table 1:** Results of linearity.

Theoretical Concentration (mg/mL)	Mean Peak Area (mAU.min) ± SD (n = 3)	Calculated Concentration (mg/mL)
0.8	24.35 ± 0.08	0.79 ± 0.03
0.9	28.19 ± 0.03	0.90 ± 0.01
1.0	31.60 ± 0.11	0.99 ± 0.04
1.1	35.49 ± 0.15	1.10 ± 0.05
1.2	38.35 ± 0.08	1.19 ± 0.03

**Table 2:** Results of precision.

Concentration (mg/mL)	Intraday Precision		Interday Precision	
	Mean Peak Area ± SD (n = 3)	RSD (%)	Mean Peak Area ± SD (n = 6)	RSD (%)
0.85	25.5 ± 0.1	0.2	25.5 ± 0.1	0.3
1.0	30.4 ± 0.1	0.2	30.6 ± 0.1	0.2
1.15	35.5 ± 0.1	0.3	35.3 ± 0.1	0.3

**Table 3:** Results of accuracy.

Theoretical Concentration (mg/mL)	Calculated Concentration (mg/mL)	Recovery (%)	RSD (%)
0.80	0.81 ± 0.03	101.5 ± 0.3	0.3
0.90	0.90 ± 0.05	100.3 ± 0.9	0.9
1.0	1.01 ± 0.03	100.8 ± 0.3	0.3
1.10	1.10 ± 0.03	100.3 ± 0.3	0.3
1.20	1.21 ± 0.04	100.8 ± 0.3	0.3

**Table 4:** Extraction recovery of 1% CLA cream according to the solvent and exposition time.

Extraction Solvent	Times (h)	Recovery (%)
Acetone	1	41
	3	46
Acetonitrile	1	77
Methanol	1	69
	3	80

**Table 5:** Forced degradation of 1% CLA cream.

Stress Condition	% CLA Remaining			Retention Time of Degradation Product (min)
	H0	H1	H48	
Acidic stress (HCl 5 M, 60 °C, 48 h)	51	0	0	2.7–4.4
Alkaline stress (NaOH 5 M, 60 °C, 48 h)	97	97	97	2.9
Oxidative stress (3%, H_2_O_2_, 60 °C, 48 h)	92	14	0	2.8–4.0–4.7–6.0

**Table 6:** Composition of 1% CLA cream.

Ingredients	Amount for 100 g
Clarithromycin lactobionate	1.49 g (equivalent to 1 g of clarithromycin base)
Transcutol^®^	10 g
Ascorbic acid	0.1 g
Pentravan^®^	Adjust to 100 g
Sodium hydroxide	pH adjusted to 7.3 ± 0.2

## Data Availability

Manuscript contains all key data.
